# Three-dimensional mouse cochlea imaging based on the modified Sca/eS using confocal microscopy

**DOI:** 10.1007/s12565-023-00703-z

**Published:** 2023-02-11

**Authors:** Shinji Urata, Shigeo Okabe

**Affiliations:** 1 Department of Otolaryngology, Graduate School of Medicine, University of Tokyo, Tokyo, 113-0033 Japan; 2 Department of Cellular Neurobiology, Graduate School of Medicine, the University of Tokyo, Tokyo, Japan

**Keywords:** Stria vascularis, Cochlea, Optical tissue clearing, Sca*l*eS, Macrophage

## Abstract

The three-dimensional stria vascularis (SV) and cochlear blood vessel structure is essential for inner ear function. Here, modified Sca/eS, a sorbitol-based optical-clearing method, was reported to visualize SV and vascular structure in the intact mouse cochlea. Cochlear macrophages as well as perivascular-resident macrophage-like melanocytes were detected as GFP-positive cells of the CX3CR1^+/GFP^ mice. This study’s method was effective in elucidating inner ear function under both physiological and pathological conditions.

**Supplementary Information:**

The online version contains supplementary material available at 10.1007/s12565-023-00703-z.

## Introduction

**Fig. 1:**
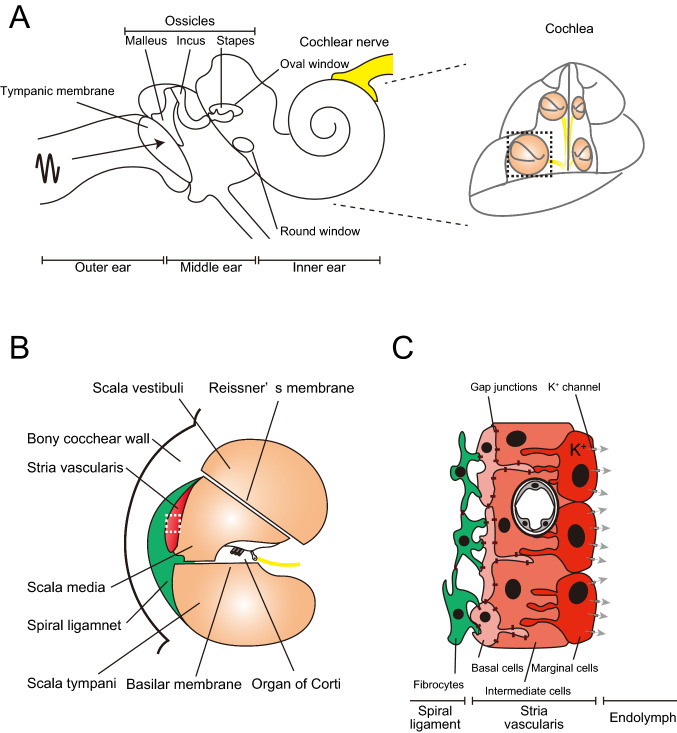
Auditory pathway anatomy. **A** Sound wave is transferred to the tympanic membrane. Stimulation is amplified by three tiny bones (“ossicles”) and transferred to the inner ear through the oval window to the round window in the cochlea. Stimulation vibrates the basilar membrane in the cochlear duct (pale orange) and electrical stimuli converted in the Corti organ are transferred to the brain through the cochlear nerve (yellow). **B** Anatomical structures of the cochlear duct (black dashed squared in **A**). Membrane structures, including cochlear duct (pale orange), stria vascularis (red), spiral ligament (green), organ of Corti, and cochlear nerve (yellow), are covered with bone. **C** A schematic compartmental model of the lateral cochlear wall (white dashed squared in **B**). Stria vascularis (SV) consists of three cellular components: marginal (red), intermediate (orange), and basal (pink). Potassium-regulating channels are located in the marginal cells (arrows, flow of K^+^ ions). Gap junctions are expressed in the basal cells and connect to the intermediate cells and fibrocytes in the spiral ligament (green)

The cochlea is a sound-receptive sensory organ located in the temporal bone. Sound vibrations to the tympanic membrane are transmitted to the inner ear through the middle ear ossicles (Fig. 1A). The sensory epithelium formed by hair cells convert sound stimuli into electrical signals and the signal is transferred to the auditory cortex. Lymph fluid generated by nonsensory epithelium cells fills the cochlea and regulates cochlear function (Fig. 1B). Endolymph is produced in the stria vascularis (SV) in the bony cochlear wall (Tasaki and Spyropoulos 1959). Stratified epithelium in the SV consists of three cell types: marginal, intermediate, and basal (Fig. 1C). K^+^ transporter expressed on the marginal cells is essential in maintaining endolymphatic high potential (Nin et al. 2008). Intermediate cells are pigment-containing cells classified as a unique macrophage type, such as perivascular-resident macrophage-like melanocyte (PVM/Ms) (Zhang et al. 2012). Gap junctions connect basal cells in the SV to the fibrocytes in the spiral ligament (Kikuchi et al. 1995). The previous SV studies have elucidated cochlear function using primarily electrophysiological analysis. The SV cell group ultrastructure has been visualized using an electron microscopy. However, wide-field imaging of the lateral cochlear wall, including SV, spiral ligament, and the bony cochlear wall, has not been performed because of its anatomic location. The cochlea is covered with bone and has a hollow structure lined with a membrane. For physiological analysis of both sensory and nonsensory epithelium cells, traditional histology and immunohistochemistry should be optimized for sample sectioning. Alternatively, whole-mount surface preparation has been performed (Mizushima et al. 2017; Fujimoto et al. 2017).

Optical tissue clearing enables structural visualization and molecular information acquisition from large-volume tissues. Optical tissue-clearing methods combined with light-sheet or two-photon microscopy have enabled structural acquisition of the rodent’s sensory epithelium (Toulemonde et al. 2022; Hutson et al. 2021; Tinne et al. 2017; Brody et al. 2020; Moatti et al. 2022, 2020; Nolte et al. 2017; Risoud et al. 2017; Malfeld et al. 2021; Keppeler et al. 2021). However, an optimized method using confocal microscopy to analyze the intact rodent’s cochlea has not yet been developed (Wrzeszcz et al. 2013; MacDonald and Rubel 2008, 2010).

In this study, modified Sca/eS was described to enables us to visualize the SV structures and blood vessels and the immune cells, such as cochlea macrophages and PVM/Ms, in the intact mouse cochlea.

## Materials and methods

### Sample preparation

Mouse husbandry, anesthesia, and euthanasia conformed to related regulations of the government and the institutional guidelines. All animal studies are reviewed and approved by the Animal Care and Use Committee of the Graduate School of Medicine, the University of Tokyo. This study used male and female 8–12 week-old C57BL/6 or CX3CR1 mutant mice (Steffen et al. 2000). The mice’s CX3CR1 was replaced with GFP. Therefore, heterozygous CX3CR1^+/GFP^ mice were used on the C57BL/6 background. CX3CR1 is the specific receptor for the chemokine fractalkine (CX3CL1) and expressed in monocytes, macrophages, microglia, subsets of NK and dendritic cells, and active T cells (Steffen et al. 2000). In the mouse cochlea, CX3CR1 is expressed in resident macrophages, perivascular macrophages, and infiltrating monocytes (Hough et al. 2022). Animals were anesthetized with a ketamine−xylazine mix (ketamine, 100 mg/kg; xylazine, 20 mg/kg). After euthanasia, mice were perfused transcardially with 4% paraformaldehyde (PFA) in phosphate-buffered saline (PBS). Temporal bones were extracted and the samples were incubated in 4% PFA in PBS at 4 °C for 12 h. Decalcification was performed by incubating samples for 72 h in 500 mM EDTA in PBS at 37 °C and terminated by washing samples several times with PBS. Subsequently, the inner ear was dissected from the temporal bone under a stereo microscope.

### Transcardial vascular staining

Before transcardial perfusion−fixation, anesthetized mice were injected with 50 µl of 1 mg/ml *Lycopersicon esculentum* lectin conjugated to DyLight649 (DL-1178, VectorLabs) from the tail vein. After 5 min, mice were transcardially perfused with 4% PFA with PBS (Battistella et al. 2021).

### Labeling with small molecules

Samples were washed with PBS containing 0.1% Triton X-100 for 30 min with continuous rocking at 40 rpm. Subsequently, samples were incubated for 2 h in a solution containing 100 nM small molecules (rhodamine-conjugated phalloidin, Molecular Probes) with appropriate dilutions at 37 °C. Finally, small molecules were removed by washing for 1 h with PBS containing 0.1% Triton X-100, with continuous rocking at 40 rpm.

### Refractive index (RI) matching

Modified Sca/eS were adopted, as a tissue-clearing method, according to the previous report (Urata et al. 2019a, b). Briefly, samples were washed three times by PBS containing 0.1% Triton X-100 for 10 min after small molecules staining. Samples were placed in a chamber with the RI matching solution, covered by a coverslip, and imaged by a confocal microscope. RI matching solution consisted of 4 M urea, 60% (w/v) d-sorbitol, and 0.1% (w/v) Triton X-100 in PBS (pH 7.1).

### Image acquisition

Fluorescent images were performed on a confocal microscope system (A1R, Nikon, Japan) with a 20 × water immersion objective lens (NA = 0.95). GFP, rhodamine phalloidin, and DyLight649 were excited with 488, 564, and 635 nm, respectively. A chamber containing the sample was filled with the RI matching solution, covered by a glass coverslip, and placed under objective lens. Single horizontal image sizes were set to 512 × 512 with pixel sizes of 1.23 × 1.23 μm and z-spacing of 2 μm. The ImageJ software (National Institute of Health) was used to perform image processing and Imaris (Bitplane) and NIS-Element AR (Version 4.51, Nikon) was used to perform three-dimensional rotation.

## Results

**Fig. 2:**
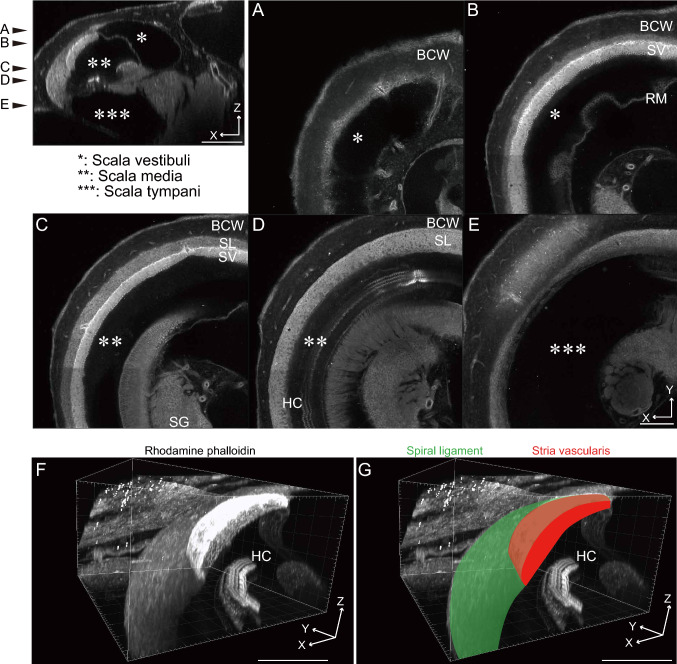
Three-dimensional structure of cytoskeleton labeled with F-actin. The cochlear duct cross-section is shown in the left top panel. Scala vestibule (single asterisk), scala media (double asterisk), and scala tympani (triple asterisk) are visualized in the cochlear duct. The two-dimensional images at several planes (**A**–**E**) are shown. The three-dimensional lateral cochlear wall is reconstructed and the stria vascularis (red) and spiral ligament (green) are clearly identified (**F**, **G**). *BCW* bony cochlear wall, *HC* hair cell, *RM* Reissner’s membrane, *SG* spiral ganglion, *SL* spiral ligament, *SV* stria vascularis. Scale bar, 200 μm

F-actin-labeled cochlea was imaged from the apical to the basal portion along the cochlear modiolus. Since F-actin protein is a major cytoskeleton component, all cochlear cells can be identified as rhodamine phalloidin-positive cells. Blood vessels toward the bony cochlear wall (arrows in Fig. 2A) and Reissner’s membrane structure (Fig. 2B) were observed. Borders of the lateral wall components were apparent (Fig. 2C). The three-dimensional image was reconstructed based on the two-dimensional images (Fig. 2F). The SV and spiral ligament’s three-dimensional structure were visualized, thus the SV and spiral ligament borders were recognized (Fig. 2F, G).

**Fig. 3:**
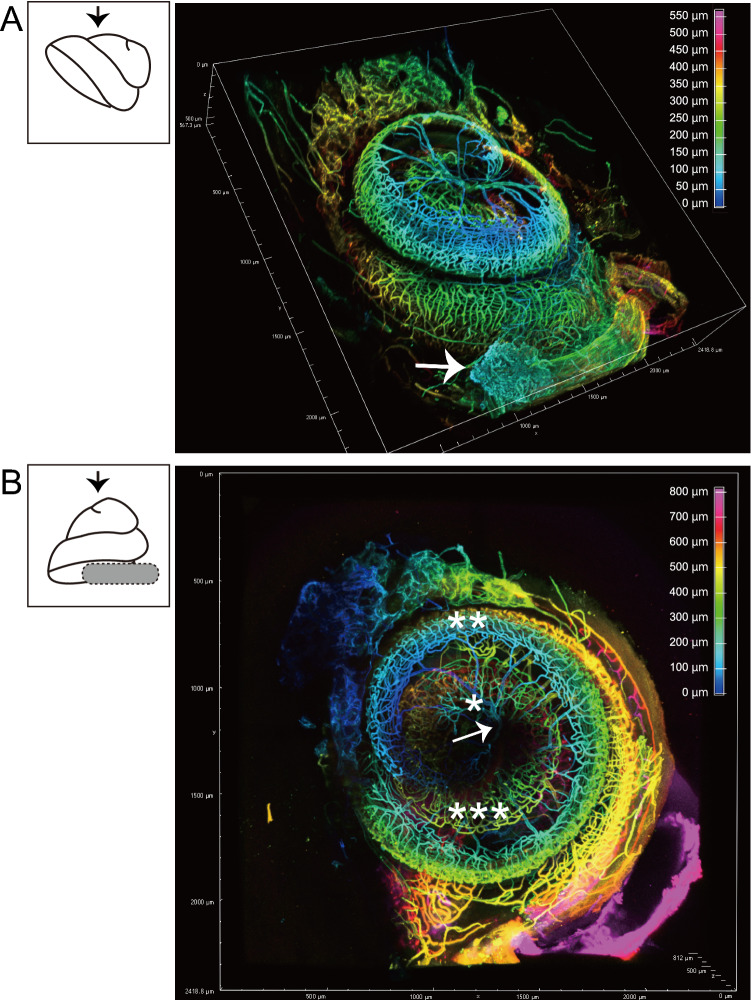
Vasculature of the mouse cochlea labeled by lectin. **A** The cochlea is placed on the glass slide. Images are acquired in parallel with the bony cochlear wall of the surface side (black arrow). Cochlear vasculature is widely visualized. Stapedial vessels on the surface of the cochlear bony wall are also observed (white arrow). The color bar shows the depth from the surface plane. **B** A pedestal (gray ellipse) is placed so that the axis of the cochlear center is aligned perpendicular to the glass slide. Images are acquired along the modiolus from the apical portion (black arrow). Radiating arterioles (white arrow), main cochlear artery (single asterisk), lateral wall vessels (double asterisk), and spiral vessels (triple asterisk) are visualized. The color bar shows the depth from the surface plane

Then, the vascular structure in the cochlea was investigated by injecting the *Lycopersicon esculentum* lectin conjugated to DyLight649 under anesthesia (Supplemental Movie 1). The stapedial artery, which passes through the stapes (arrow in Fig. 3A), can be identified, as well as cochlear blood vessels (Fig. 3A). In addition, the image along the modiolus enabled us to visualize the radiating arterioles (arrow in Fig. 3B) from the main cochlear artery in the modiolus (single asterisk in Fig. 3B) to the vessels in the lateral wall (double asterisk in Fig. 3B) and spiral vessels (triple asterisk in Fig. 3B).

**Fig. 4:**
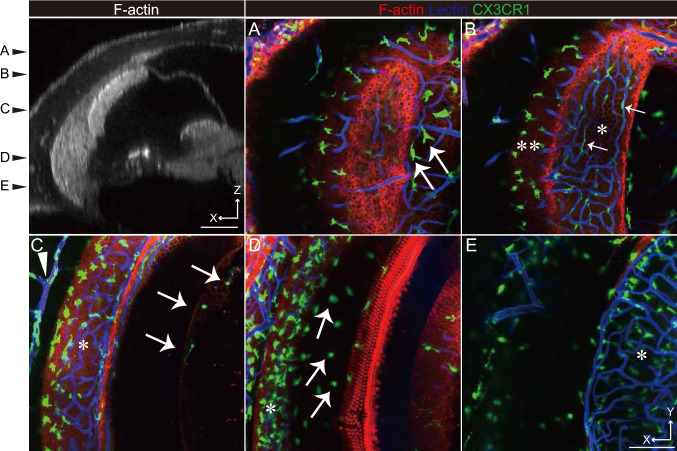
Macrophage distribution in the cochlea. The cochlear duct cross-section labeled with F-actin is shown in the left top panel. The two-dimensional cochlear images of the CX3CR1 mouse labeled with rhodamine phalloidin (red) and lectin (blue) at several planes (**A**–**E**) are shown. GFP is widely expressed in the cochlea (**A**–**E**) and various macrophage shapes, dendritic-to-amoeboid (arrows in **A**, **E**), ramified (arrows in **B**), and amoeboid (arrows in **D**), are expressed in the stria vascularis (asterisk in **B**), spiral ligament (double asterisk in **B**, single asterisk in **D**), spiral limbus (single asterisk in **E**), and cochlear bony wall (arrowhead in **C**). Scale bar, 100 μm

Macrophages were widely present in the cochlea. Nevertheless, the role of macrophages is still enigmatic (Hough et al. 2022). Its activation is characterized by changes in macrophage morphology, mediator expression, and distribution. Ramified macrophages have long dendritic processes with small cell bodies. In contrast, amoeboid macrophages are flat-shaped. GFP-positive cells were identified as macrophages (Fig. 4, Supplemental Movie 2). Ramified, amoeboid, and dendritic-to-amoeboid macrophages were located in the SV (arrows in Fig. 4B), basilar membrane (arrows in Fig. 4D), and bony cochlear wall on the scala vestibuli (arrows in Fig. 4A), respectively. Ramified macrophages were aligned to SV blood vessels. The perivascular macrophage is located in the intermediate layer because they were above the marginal cell layer (single asterisk in Fig. 4B). Additionally, macrophages were identified on Reissner’s membrane and a lectin-labeled blood vessel in the bony cochlear wall was visualized (arrows in Fig. 4C). Various macrophage types seemed to closely occupy the lower part of the spiral ligament (asterisk in Fig. 4D) compared to those of the upper (double asterisk in Fig. 4B) and middle (asterisk in Fig. 4C) parts. The cochlear macrophage shape in the spiral limbus was dendritic-to-amoeboid and located adjacent to spiral vessels (single asterisk in Fig. 4E).

## Discussion

The optical tissue-clearing method, modified Sca/eS, was demonstrated to enable structural visualization of the intact mouse cochlea’s SV and blood vessels. Furthermore, confocal microscopy could widely observe GFP expressed in mouse cochlea macrophages.

A complete understanding of the cochlear function relies on an accurate description of the three-dimensional cochlear structure. The components required for sound perception are covered by the temporal bone and cochlear bony wall. Therefore, traditional protocols, such as thin sectioning and whole-mount surface preparation, have been performed to understand cochlear morphology. Those protocols are rigidly established and highly reproducible. However, three-dimensional cellular distribution in the SV and Reissner’s membrane is still an enigma because the field of view is highly restricted in these conventional techniques.

The cochlea’s F-actin-stained cellular structure was described using the modified Sca/eS (Fig. 2). This method enabled three-dimensional structural visualization of the cochlea. The entire volumes of the organ of Corti, SV, and spiral ligament could be measured by tracing the targeted area (Supplementary Fig. 1). This method is likely to be an alternative tool for researching Meniere’s disease. Based on the clinical study with high-resolution contrast-enhanced magnetic resonance imaging, endolymphatic hydrops of the scala media (double asterisk in Fig. 2) have been considered the primary pathology of Meniere’s disease (Niyazov et al. 2001). Furthermore, in vivo and in vitro animal studies support this hypothesis (Egami et al. 2013; Kakigi et al. 2020). Nevertheless, a complete scala media survey has been difficult because the membrane structure, such as Reissner’s membrane and basilar membrane, are transformed due to fixation and/or dissection, as well as image acquisition plane perpendicular to the modiolus.

Recently, in vivo live cochlear imaging using two-photon microscopy revealed that the gentamicin transporter, expressed in the SV and involved in the blood−labyrinthine−barrier function, protects against gentamicin-induced hearing loss, which causes drug-induced hearing loss in a significant patient population (Kim and Ricci 2022). Including drug-induced hearing loss, SV is an important component because sound exposure, which causes noise-induced hearing loss, decreases SV blood flow (Burwood et al. 2020). Previous reports have revealed cochlear blood vessel details. However, complete morphology of intact cochlea has not been acquired (Nomura and Hiraide 1968; Axelsson 1988; Jiang et al. 2019). In recent advancements in optical tissue clearing, the intact mouse brain’s blood vessel structure cleared by CLARITY (Giovanna et al. 2018) and 3DISCO (Lugo-Hernandez et al. 2017) were visualized. Furthermore, unbiased and scalable vasculature quantification of cleared mouse brains was developed using machine learning (Todorov et al. 2020). Visualization of cochlear blood vessels labeled with lectin (Fig. 3, Supplemental Movie 1) was demonstrated. The method will quantify the cochlea’s angioarchitecture under various conditions, such as age-, noise-, and drug-induced hearing loss.

Accumulating information about macrophages in the cochlea is essential to understand the inner ear’s immune system. Macrophages are pervasive in the steady-state cochlea: SV, spiral ligament, spiral ganglion, and basilar membrane (Hirose et al. 2005). Macrophages in the cochlea responds against stresses derived from noise exposure and ototoxic drug and its morphology is transformed by noise-, drug- and age-induced hearing loss (Frye et al. 2017). In animal studies, macrophages in Reissner’s membrane have been derived only under stressed conditions (Sautter et al. 2006). However, macrophages in Reissner’s membrane were observed in humans suffering from life-threatening posterior cranial fossa meningioma (Liu et al. 2021). Recently, CX3CR1-positive cells were recognized in Reissner’s membrane of the quiescent mouse cochlea by using two-photon microscopy (Bae et al. 2021). This study’s result is consistent with this report (arrows in Fig. 4C, Supplementary Movie 2).

Previous reports have revealed two macrophage types in the lateral cochlear wall: cochlear macrophage in the spiral ligament and PVM/Ms in the SV (Fujioka et al. 2014). PVM/Ms is a melanin-positive macrophage and maintains blood−labyrinthine barrier integrity (Zhang et al. 2012). Moreover, based on their location and morphology, previous reports proposed that PVM/Ms can be classified as pericytes and intermediate cells. PVM/Ms are macrophages bearing dendritic processes located adjacent strial capillaries (Shi 2010; Neng et al. 2013). All CX3CR1-positive cells in the intermediate cell layer were found on blood vessels (arrows in Fig. 4B, Supplementary Movie 2).

The imaging depth of conventional confocal imaging is limited to approximately 100 μm, prohibiting wide-field imaging with a single-cell resolution of the cochlea (Nwaneshiudu et al. 2012). Optical access to cell properties is an effective tool for cell biology. Various optical tissue-clearing methods, including Sca/e (Hama et al. 2011), CLARITY (Chung and Deisseroth 2013), CUBIC (Susaki et al. 2014), and 3DISCO (Ertürk et al. 2012), have enabled mouse brain structural and molecular information acquisition. Recently, other advanced techniques optimized for wide-field imaging (Susaki et al. 2015; Murakami et al. 2018) and hard tissue containing extracellular matrix (Greenbaum et al. 2017; Wang et al. 2019; Pan et al. 2016) were reported. Using two-photon microscopy, both organic- (MSBB (Hutson et al. 2021): methyl-salicylate and benzyl benzoate, iDISCO (Moatti et al. 2020)) and hydrophilic-solution-based clearing methods (Urata et al. 2019b) enabled us to visualize gerbil (Hutson et al. 2021), porcine (Moatti et al. 2020), and mouse (Urata et al. 2019b) sensory epithelium cells in the cochlea. Even though optimized MSBB is an organic-solution-based clearing method, confocal microscopy is insufficient to visualize all components of intact rodent’s cochlea (Risoud et al. 2017; Malfeld et al. 2021; Wrzeszcz et al. 2013; MacDonald and Rubel 2008, 2010).

The imaging method described in this study has several limitations. GFP and *Lycopersicon esculentum* lectin fluorescence conjugated to DyLight649 were sufficient for detection by single-photon excitation. However, rhodamine phalloidin fluorescence was weaker and laser intensity adjustment was necessary for deep tissue imaging. Furthermore, deep inside the cochlea, fine structures (stereocilia) found on hair cell surfaces were not visualized. Improvement in the labeling method may overcome this difficulty, but the current immunostaining techniques combined with conventional confocal microscopy were insufficient. Therefore, a 2-way imaging protocol was developed according to the previous reports (Supplementary Fig. 2). The method supported image acquisition of mouse cochlea all along its imaging depth. Recently, gerbil (Toulemonde et al. 2022) and guinea pig (Brody et al. 2020) sensory epithelium have been visualized by nontoxic organic compound ethyl cinnamate (ECi) with two-photon microscopy. Refractive index (RI) of ECi (1.558) is similar to DBE (1.562) which was used as the mounting solution of organic-solution-based-clearing method. Therefore, ECi seems to be useful for intact cochlear imaging by confocal microscopy.

## Supplementary Information

Below is the link to the electronic supplementary material.

Supplementary Movie 1 Visualization of the intact blood vessels of the mouse. The structure of the blood vessels in the cochlea was labeled with lectin and the image was shown from the apical to the basal portions (AVI 2804 KB)

Supplementary Movie 2 Three-dimensional reconstruction of the cochlea duct. The macrophages (green), cytoskeleton (red), and blood vessels (blue) are identified, and the structures are three-dimensional reconstructed. XY plane in Figure 4 is shown from the Reissner’s membrane level to the basilar membrane. Subsequently, the structures are presented in the XZ plane (AVI 52611 KB)

Supplementary Figure 1 Three-dimensional rendering of the cochlear components. The organ of Corti (green), stria vascularis (magenta), and spiral ligament (blue) are reconstructed by a series of two-dimensional traced images (lower right). Supplementary Figure 2 Application for whole cochlea imaging by confocal microscopy. The sample’s apical half was imaged by single-photon (1p) microscopy, the sample was then inverted, and the basal half was imaged. Green: CX3CR1-GFP, Red: F-actin, Cyan: lectin. (DOCX 260 KB)
