# Supplementary material for: Three-dimensional mouse cochlea imaging based on the modified Sca/eS using confocal microscopy
Source: Anat Sci Int. 2023 Feb 11;98(3):309–17. doi: 10.1007/s12565-023-00703-z (PMC10256671; doi:10.1007/s12565-023-00703-z)
Supplement: Supplementary file 1 [file 12565_2023_703_MOESM3_ESM.docx]

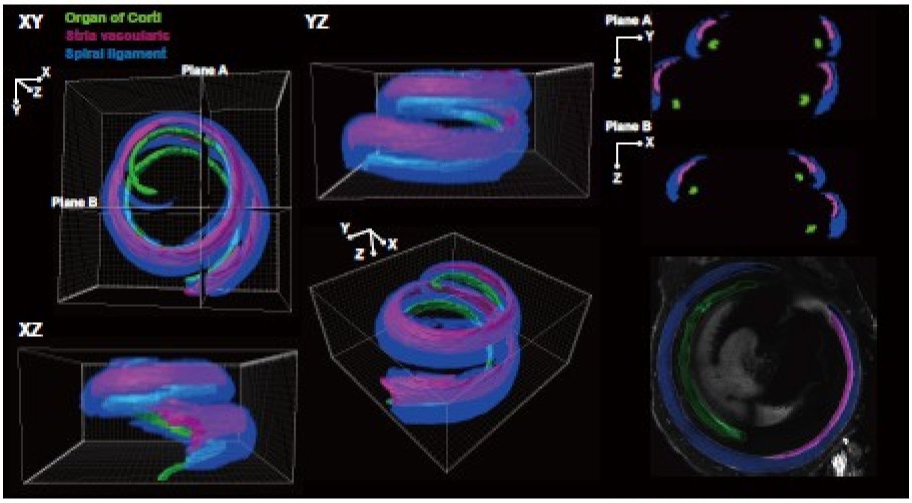

Supplementary Figure 1

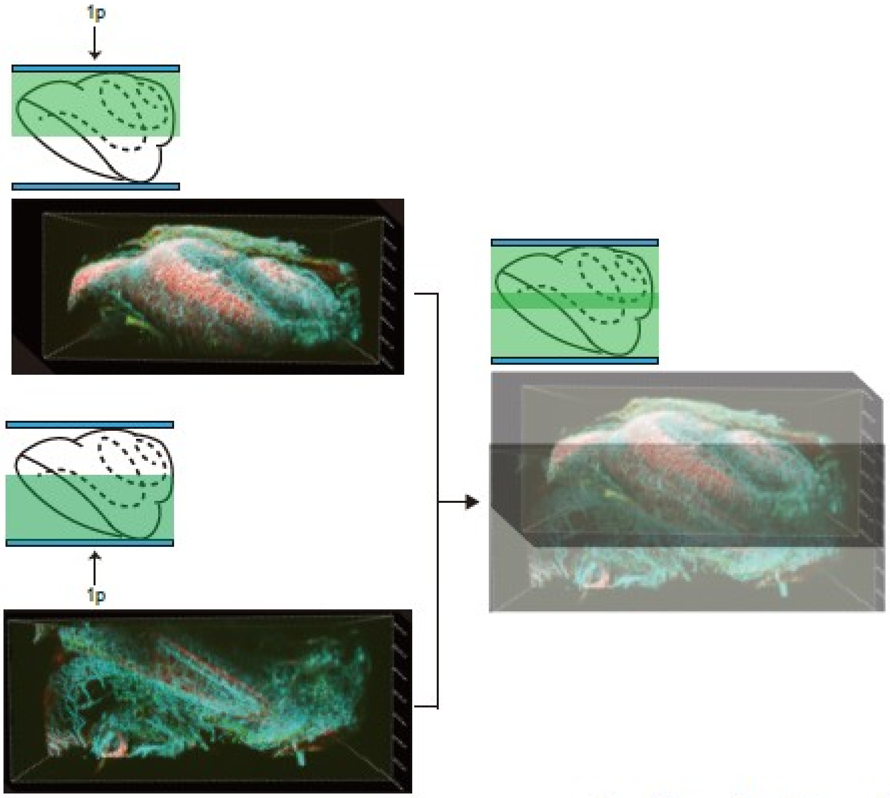

Supplementary Figure 2
